# Delivery of written and verbal information on healthcare-associated infections to patients: opinions and attitudes of a sample of healthcare workers

**DOI:** 10.1186/s12913-017-2021-x

**Published:** 2017-01-23

**Authors:** Marco Bo, Viola Amprino, Paola Dalmasso, Carla M. Zotti

**Affiliations:** Department of Public Health and Paediatrics Sciences, University of Turin, via Santena 5/bis, Turin, 10126 Italy

**Keywords:** Access to Information, Hospitals, Cross-infections

## Abstract

**Background:**

Patients education is considered a valuable mean to prevent and control healthcare-associated infections (HAIs). This cross-sectional study aims to assess declared practices of healthcare workers (HCWs) regarding the delivery of information about HAIs to patients.

**Methods:**

A 14-item multiple-choice questionnaire was designed to assess the attitudes and declared practices of HCWs (physicians, nurses and nursing assistants). Between October 2012 and October 2013, we surveyed a sample of HCWs from 4 acute hospitals in Piedmont (North-western Italy). Written information was available at three hospitals (A, B and C) and verbal information at the last one (hospital D).

**Results:**

We surveyed 288 HCWs (79 physicians, 124 nurses and 85 healthcare assistants). At hospital A, B and C, 128 (71.6%) HCWs declared that written information was usually delivered to any patient and 145 (66.5%) that nurses usually delivered it. Only 42 (26.3%) of them – 97.6% nurses –declared that they usually delivered written information to patients. Among all surveyed HCWs, 210 (72.9%) declared that patients also receive verbal information on HAI – mainly by nurses (70.8%) and physicians (50%) – but only 88 (29,2%) – 23.8% physician and 48.8% nurses – declared that they usually informed patients. Finally, 83 (27.7%) HCWs believed that they should decide whether or not to deliver information to patient case by case.

**Conclusions:**

A formal policy requiring to deliver written information is most likely not enough to induce HCWs to better inform patients about HAIs. Health Trusts might introduce more target actions to reinforce HCWs’ practices, such as training and internal auditing.

**Electronic supplementary material:**

The online version of this article (doi:10.1186/s12913-017-2021-x) contains supplementary material, which is available to authorized users.

## Background

Healthcare-Associated Infections (HAIs) are the fourth leading cause of disease in the industrialized countries, where they are a major cause of morbidity and mortality among hospitalised patients [1]. Indeed, according to the last European point prevalence survey, on any given day, about 80,000 (5.7%) patients in European acute care hospitals had at least one HAI [2]. At the same time, it is plausible that at least 20% of nosocomial infections are preventable [3]. Thus, the surveillance of HAIs and the development of appropriate policies for infection control have a high priority both in US and in EU [4].

Many studies have shown that multi-modal interventions can significantly reduce the rate of nosocomial infections, mainly bloodstream infections and urinary tract infections [3]. Multi-modal action often involves both the patients and the caregivers; in addition well-educated and informed patients can collaborate in the prevention of HAIs. For this reason, efforts to increase patients’ awareness of HAIs was recently recommended.

In 2004, the French Ministry of Health recommended delivering general information on HAIs to every inpatient at admission through a recovery guidebook. Patients should also receive information tailored to the treatments delivered and to their individual risks of infection during their recovery, with special attention to patients who had acquired an HAI [5].

In 2009, the Council of Europe also recommended the delivery of general information on HAIs to every inpatient [6]. In the same period, in North-Western Italy, the Piedmont Regional Health Authority approved a policy recommending the delivery of written information on HAIs to every patient at admission to regional hospitals [7]. The availability of written material on HAIs for every inpatient was also included among the regional indicators for the prevention and control of HAIs.

Despite European and national recommendations, few data are available on the delivery of information on HAIs to patients. Only a French study showed that healthcare workers were not prone to inform their patients about the risk of contracting an HAI and no data are available on the Italian context [8]. Thus, we performed a cross-sectional survey to describe the attitudes and behaviours of healthcare workers on the delivery of information about HAIs.

## Methods

We conducted a cross-sectional survey at 4 hospitals in Piedmont from October 2012 to October 2013. Information on HAIs was delivered through a recovery guidebook at one hospital (hospital A), through an informational leaflet at two hospitals (hospitals B and C), and no written information on HAIs was delivered at the last hospital (hospital D). Four to 6 wards were identified at every hospital and selected among internal medicine, specialised medicine (cardiology, oncology, haematology, respiratory disease and gastroenterology) and surgery units. We asked to complete the questionnaire to all healthcare workers (physicians, nurses and nursing assistants) who were attending to patients at any of the selected wards during the days of administration of the survey. We conducted the survey in different days of the week and at different hours of the day in each ward, to interview most of the healthcare workers employed at each of the units.

We designed a 14-item questionnaire to evaluate whether healthcare workers were aware of the policy in force at the hospital where they worked, on the ways such a policy was applied in the ward in which they operated and to examine their experiences and opinions about the information provided to patients about HAIs (see Additional file 1). Three of the questions were adapted from questions used in a previous survey [9]; the others were tailored to investigate whether HCWs applied the regional policy. Each question provided 1 to 4 closed answers, depending on the argument. Two resident doctors administered the questionnaires.

We validated the questionnaire through a survey of 30 patients, randomly selected at two of the hospitals. Then, Cohen's kappa was used to calculate the percentage of overall agreement on the questions investigating the HCWs’ awareness on the policy in force (Q1-Q5, K = 0.77; Q1-Q10 answer 1, K = 0.8). The way both to ask questions and to suggest their closed answers and to give explanations to the surveyed healthcare workers were discussed with the resident doctors during the pilot study.

The study was approved by the Ethics Committee of the University of Torino. Formal written authorisations to conduct the study were obtained by the administration of any participating hospital. An informational leaflet explaining the aim and the characteristics of the study was delivered to every participant. All participants gave their written consent to take part in the study.

### Statistical analysis

Data were expressed as counts and percentages. Univariate analyses was performed using a chi-squared test or Fisher’s exact test, as appropriate, to verify the existence of significant differences by qualification and by hospital.

All tests were two tailed, and the statistical significance level was set at 0.05.

All of the analyses were performed using STATA SE 12.1.

## Results

We surveyed 288 healthcare workers at four hospitals in the Piedmont: 79 physicians, 124 nurses and 85 nursing assistants. About 70% of respondents were females, mainly represented by nurses and nursing assistants (85%). No significant differences were found by gender, age, qualification or ward among the hospitals. The characteristics of the sample are reported in Table 1.

**Table 1:** Characteristics of the sample

	Hospital A (*n* = 56)	Hospital B (*n* = 73)	Hospital C (*n* = 89)	Hospital D (*n* = 70)	Total (*n* = 288)	*p*-value
Gender						
Female	40 (71.4)	44 (60.3)	69 (77.5)	49 (70.0)	202 (70.1)	0.12
Age						
23–35 years	17 (30.4)	13 (17.8)	26 (29.2)	23 (32.8)	79 (27.4)	0.15
36–55 years	35 (62.5)	49 (67.1)	58 (65.2)	38 (54.3)	180 (62.5)	
> 56 years	4 (7.1)	11 (15.1)	5 (5.6)	9 (12.9)	29 (10.1)	
Qualification						
Physician	10 (17.9)	27 (37.0)	27 (30.4)	15 (21.4)	79 (27.4)	0.20
Nurse	26 (46.4)	25 (34.2)	39 (43.8)	34 (48.6)	124 (43.1)	
Nursing assistant	20 (35.7)	21 (28.8)	23 (25.8)	21 (30.0)	85 (29.5)	
Ward						
Internal Medicine	15 (26.8)	14 (19.2)	29 (32.6)	28 (40.0)	86 (29.9)	0.07
Specialised Medicine	14 (25.0)	29 (39.7)	29 (32.6)	15 (21.4)	87 (30.2)	
Surgery	27 (48.2)	30 (41.1)	31 (34.8)	27 (38.6)	115 (39.9)	

In hospitals where any written informational materials on HAIs were available (A, B and C), 156 (72%) HCWs were informed about the policy and almost all of them were able to describe the type of written material that ought to be delivered to the patients (Q1 and Q2, Table 2).

**Table 2:** Answers given by healthcare workers stratified by hospital

Questions	Question asked to the healthcare workers	Hospital A (*n* = 56)	Hospital B (*n* = 73)	Hospital C (*n* = 89)	Hospital D (*n* = 70)	*p*
Q1	Do you know whether any WRITTEN information on Healthcare-Associated Infections is distributed to all inpatients at admission?					
	- Yes	37 (66.1)	51 (69.9)	68 (76.4)	0 (−)	<0.001
	- No	19 (33.9)	22 (30.1)	21 (23.6)	70 (100)	
	If yes:					
Q2	▪ What kind of informational material is distributed in this hospital?					
	- an informational leaflet	0 (−)	50 (98.0)	68 (100.0)	--	*<0.001*
	- an admission guidebook	37 (100.0)	1 (2.0)	0 (−)	--	
Q3	▪ Is written informational material on Healthcare-Associated Infection available in the ward in which you work?					
	- Yes	35 (94.6)	51 (100.0)	68 (100.0)	--	*0.06*
	- No	2 (5.4)	0 (−)	0 (−)	--	
Q4	▪ Is written informational material distributed to any inpatient able to read it at admission?					
	- Yes	28 (75.7)	32 (62.7)	68 (100.0)	--	*<0.001*
	- No	8 (21.6)	18 (35.3)	0 (−)	--	
	- I don’t remember	1 (2.7)	1 (2.0)	0 (−)	--	
Q5	▪ Who usually delivers informational material on Healthcare-Associated Infections to inpatients?					
	-physicians	0 (−)	0 (−)	0 (−)	--	*-*
	-nurses	35 (97.2)	48 (100.0)	62 (91.2)	--	*0.06*
	- nursing assistants	8 (22.2)	3 (6.3)	8 (11.8)	--	*0.10*
Q6	Do you know whether inpatients receive any VERBAL information on Healthcare-Associated Infections in the ward in which you work?					
	- Yes	41 (73.2)	64 (87.7)	70 (78.7)	35 (50.0)	<0.001
	- No	15 (26.8)	9 (12.3)	19 (21.3)	35 (50.0)	
Q7	If yes, who usually gives verbal information on HAIs to inpatients?					
	- physicians	24 (58.5)	48 (75.0)	48 (70.6)	24 (68.6)	*0.35*
	- nurses	41 (100.0)	63 (98.4)	67 (95.7)	33 (94.3)	*0.40*
	- nursing assistants	7 (17.1)	10 (15.6)	39 (56.5)	24 (68.6)	*<0.001*
Q8	Do you know whether the patients’ relatives also receive any verbal information on Healthcare-Associated Infection in the ward in which you work?					
	- Yes	23 (41.1)	41 (60.3)	55 (78.6)	16 (44.4)	<0.001
	- No	14 (25.0)	3 (4.4)	6 (8.6)	1 (2.8)	
	- only when the patient cannot read or understand information	13 (23.2)	5 (7.4)	6 (8.6)	10 (27.8)	
	- only when the patients has acquired an infection	6 (10.7)	19 (27.9)	3 (4.2)	9 (25.0)	
Q9	Have you ever been asked for information on Healthcare-Associated Infections by a patient?					
	- Never/sometimes	55 (98.2)	70 (95.9)	78 (87.6)	67 (95.7)	0.06
	- Usually/always	1 (1.8)	3 (4.1)	11 (12.4)	3 (4.3)	
Q10	In your opinion, which patients should receive information on the measures to prevent Healthcare-Associated Infections?					
	- Any inpatient	41 (73.2)	43 (58.9)	64 (71.9)	49 (70.0)	0.51
	- Only the inpatients at an increased risk of acquiring a healthcare-associated infection	0 (−)	2 (2.7)	1 (1.1)	2 (2.9)	
	- Only the patients who are infected	0 (−)	1 (1.4)	2 (2.3)	0 (−)	
	- Healthcare professionals should determine whether to give information to the patients on a case-by-case basis	15 (26.8)	27 (37.0)	22 (24.7)	19 (27.1)	

All the professionals working at hospitals B and C and the 94.6% of the ones working at hospital A reported that the informational material was available at their ward and 128 (82.1%) of them stated that it was usually distributed to any inpatient able to read it at admission (Q3 and Q4, Table 2). On the contrary, according to 21 HCWs the information materials were not usually delivered to patients: 13 (61.9%) declared that it would give a negative image to the hospital, while 8 (38.1%) stated that they have not enough time to deliver it.

When asked who usually deliver information materials on HAI, most of the professionals (from 91.2% at hospital C to 100% at hospital B) declared that nurses usually deliver it to patients (Q5). When the answers were stratified by qualification (Table 3), we found that none of the physicians and only the 2.3% nursing assistants reported usually delivering such material. On the contrary, the 54% of the nurses stated that they usually or always distribute written information to their inpatients (Q11).

**Table 3:** Answers given by healthcare workers stratified by qualification

Question	Question asked to the healthcare workers	Physicians (*n* = 79)	Nurses (*n* = 124)	Nursing assistants (*n* = 85)	p
Q1	Do you know whether any WRITTEN information on Healthcare-Associated Infections is distributed to all inpatients at admission?				
	- Yes	36 (45.6)	76 (61.3)	44 (51.8)	0.08
	- No	43 (54.4)	48 (38.7)	41 (48.2)	
	If yes:				
Q5	▪ Who usually delivers informational material on Healthcare-Associated Infections to inpatients?^a^				
	- physicians	0 (−)	0 (−)	0 (−)	*-*
	- nurses	34 (97.1)	70 (93.3)	41 (97.6)	*0.60*
	- nursing assistants	0 (−)	9 (12.0)	10 (23.8)	*0.004*
Q11	▪ Have you ever delivered it to your inpatients?^a^				
	- never/sometimes	36 (100.0)	35 (46.0)	43 (97.7)	<0.001
	- usually/always	0 (0.0)	41 (54.0)	1 (2.3)	
Q7	If inpatients receive any verbal information on Healthcare-Associated Infections in the ward you are working in, who usually gives it?				
	- physicians	40 (74.1)	48 (55.8)	56 (82.4)	0.001
	- nurses	52 (96.3)	84 (97.7)	68 (97.1)	0.88
	-nursing assistants	12 (22.6)	32 (37.2)	36 (51.4)	0.005
Q12	Have you ever given verbal information on healthcare workers to your inpatients?				
	- never/sometimes	59 (74.7)	83 (66.9)	62 (72.9)	0.44
	- usually/always	20 (25.3)	41 (33.1)	23 (27.1)	
Q9	Have you ever been asked for information on Healthcare-Associated Infections by a patient?				
	- never/sometimes	76 (96.2)	113 (91.1)	81 (95.3)	0.32
	- usually/always	3 (3.8)	11 (8.9)	4 (4.7)	
Q10	In your opinion, which patients should receive information on the measures to prevent Healthcare-Associated Infections?				
	- Any inpatient	44 (55.7)	90 (72.6)	63 (74.1)	0.01
	- Only the inpatients at an increased risk of acquiring a healthcare-associated infection	4 (5.1)	1 (0.8)	0 (−)	
	- Only the patients who are infected	2 (2.5)	0 (−)	1 (1.2)	
	- Healthcare professionals should determine whether to give the information to the patient on a case by case basis	29 (36.7)	33 (26.6)	21 (24.7)	

Q5: multiple response question

^a^Questions asked only to HCWs who declared that any informational material was delivered to inpatients in the hospital where they were working (36 physicians, 76 nurses and 44nursing assistants)

The way professionals delivered verbal information on HAIs also varied among the hospitals. Even if 210 (72.9%) of all surveyed HCWs declared that patients usually receive verbal information on HAIs, this proportion was higher where written information material was available – ranging from 73.2% at hospital A to 87.7% at hospital B – than at hospital D (50%), where only verbal information was administered (Q6, Table 2). Even in this case, almost all the professionals (from 94.3% at hospital D to 100% at hospital A) declared that nurses usually delivered verbal information (Q7), but from 58.5% (hospital A) to 75.0% (hospital B) of them also stated that physicians usually inform patients, without significant differences among the hospitals (*p* = 0.40 and *p* = 0.35 respectively). When the answers were stratified by qualification, the proportion of professionals who declared that they usually or always deliver verbal information were low among all the kind of professionals and varied among 25.3 to 33.1% (Q12, Table 3).

When we investigated single HCW’s opinion regarding the need to inform each patients on the risk of acquiring an HAI, we found out that 91 (31.6%) HCWs did not agree with the regional policy, especially among physicians. In particular, 36.7% of physicians, 26.6% of nurses and 24.7% of nursing assistants preferred to evaluate to give or not information case by case. Furthermore, 7.6% of physicians preferred only to inform patients with an HAI or at higher risk of infection. The answers are similar when stratified among the hospitals (Q10, Tables 2 and 3).

Also, HCWs disagreed regarding the choice to give information to the patients’ relatives (Q8, Table 2): while at hospital A, about a quarter of the HCWs declared that no information was given to the patients’ relatives, such a proportion was limited at the other hospitals (*p* < 0.001). Moreover, about a quarter of the respondents declared that information was only delivered to the patients’ relatives when the patients were unable to read or understand information at hospital A and D or when they had acquired an HAI at hospital B and D.

Finally, 270 (93.8%) of professionals who participated to the survey stated that patients do not usually ask questions regarding HAIs, without significant differences among qualifications (*p* = 0.32) (Q9, Tables 2 and 3).

## Discussion

This survey shows that a relevant percentage of the HCWs did not implement the regional policy requiring them to deliver written information on HAIs to all inpatients, even at hospitals where such a policy was formally adopted.

Our findings are partially comparable to ones presented in previous studies. Similar to Merle and colleagues [8], even in the present study, only a limited number of professionals reported to have personally given information on HAIs to patients, even though most of them stated that information was usually delivered to all inpatients at their ward. Also the proportions of HCWs who declared that they usually gave written or verbal information to the patients is comparable to those reported by Duclos et colleagues among HCWs interviewed during 2004 at some short stay hospitals in Paris [10]. A further research carried out in Normandy in four hospitals with psychiatric activity has shown inadequate HCW’s communications to the patients about HAIs [9].

At the same time, compared to Merle and colleagues [8], who reported that physicians were more likely than nurses to inform the patients, the present study shows the contrary. In particular, the distribution of written material on HAIs seems to be a task usually assigned only to nurses.

In our opinion, this fact may be mainly explained by organisational differences among the examined regions. Indeed, even if previous studies showed that nurses are usually more prone than physicians to follow recommendation [1], in Piedmont, the delivery of informational material on HAI is expected to be part of the tasks of the nurses who take care of the patients at their admission to the ward. Physicians most likely tell the patients about the risk of acquiring an HAI during clinical conversations preceding the acquisition of informed consent. This practice might explain why none of the physicians declared to deliver written information on HAI, while many of them stated to verbally inform patients, especially the ones working in a surgical unit.

More in general, our findings suggest that the simple launch of a policy requiring to deliver written informational material is most likely not enough to induce the HCWs to better inform their patients about the risk of acquiring an HAI.

As reported in previous studies, HCWs struggle to adopt behaviours recommended by policies and guidelines. Many factors may influence the possibility to really implement recommendations, such as workload and staff resources and the perceived inconvenience or usefulness to conform to single recommendations [1]. In our sample some of the HCWs complained about lack of time to inform patients and many of them seemed to disagreed with the regional policy. They preferred to decide whether or not to inform patients on a case by case basis or to inform only patients at higher risk of infection. Coherently, even Merle and coll. found that only approximately 17% of the interviewed professionals stated they usually gave verbal information to patients who had not acquired an HAI, while such a proportion increased to 31.6% of patients affected by an HAI [8]. Also, Leonard – who investigated the adherence to good practices among nurses caring for patients at the high risk of infection – found that approximately 95% of the oncology nurses reported that the personal risk of developing an infection and the ways to reduce it were usually discussed with the patients [11].

Also, as reported by Merle and coll., one of the main reasons why HCWs did not give information on HAI was the lack of frequent requests by the patients [8]. This result seems to be consistent with our findings because most of the professionals we interviewed, irrespective of their role, reported that the patients rarely ask for information on HAIs.

Finally, organization plays a relevant role. A strong support by the hospital management, the accessibility of local protocols and receiving specific training may improve adherence to care practices [1]. Also, HCWs seems to prefer direct ways of communication – such as face to face or phone interactions – than indirect ones, such as policies. Thus, a persistent involvement of the hospital infection prevention and control team in verbal communication regarding ward problems in the management of infections may reinforce recommendations [1, 12].

This study has some limitations. We surveyed HCWs that belonged to hospitals with different policies and we did not evaluate if the IPC teams and the ward managers took specific actions to involved HCWs in patients education. Further the sample we extracted was not randomised and we did not collect data on HCWs’ experience, such as years in practice and specific training on HAI prevention. Therefore, because the interview was trying to highlight working attitudes, corrected behaviours in line with the hospital policies may be overestimated.

## Conclusions

Even if the availability of written informational material might support patients’ education regarding HAIs, such policy might be reinforced by more focused strategies. For this purpose, it may be helpful to supply HCWs with special training on the matter, to offer auditing on the difficulties they confront in the improvement of this policy and to consider the proportion of patients receiving written information as an indicator of the quality of IPC strategies.

## Additional file

Additional file 1: Questionnaire used to conduct the survey. 14-item questionnaire designed to conduct the survey. (DOCX 17 kb)

## Abbreviations

HAI: Healthcare-associated infection
HCW: Healthcare worker
