# Supplementary material for: Delivery of written and verbal information on healthcare-associated infections to patients: opinions and attitudes of a sample of healthcare workers
Source: BMC Health Serv Res. 2017 Jan 23;17:66. doi: 10.1186/s12913-017-2021-x (PMC5259916; doi:10.1186/s12913-017-2021-x)
Supplement: Additional file 1: — Questionnaire used to conduct the survey. 14-item questionnaire designed to conduct the survey. (DOCX 17 kb) [file 12913_2017_2021_MOESM1_ESM.docx]

**Questionnaire used to conduct the survey.**

Personal code:______

Gender: M F

Age: 23-30 31-35 36-45 46-55 >55

Qualification: Physician/resident doctor Nurse Nursing assistant

Do you know whether any written information on Healthcare-Associated Infections is distributed to all inpatients at admission?

- yes
- no

If yes, what kind of informational material is distributed in this hospital?

- an informational leaflet
- an admission guidebook

Is written informational material on Healthcare-Associated Infection available in the ward in which you work?

- yes
- no

Where do we can find a copy? (describe)__________________________________________________

Is written informational material distributed to any inpatient able to read it at admission?

- yes
- no
- I don't remember

If no, why is it not distributed?

- because this kind of information may lead to anxiety
- because this kind of information may alarm patients and may deter themfrom seekinghealthcare services
- because placing an excessive emphasis on Healthcare-Associated Infections could induce patients to filemalpractice claims
- because this kind of information would give a negative image to the hospital
- because I do not have enough time to deliver it
- other (describe)___________________________________________________________________

Who usually delivers informational material on Healthcare-Associated Infections to inpatients?

- the physicianin turn at admission
- the physician who is treating the patient
- the nursein turn at admission
- the nurse who cares the patient
- nursing assistants
- another professional

Have you ever delivered it to your inpatients?

- never
- sometimes
- usually
- always

Do you know whether inpatients receive any verbal information on Healthcare-Associated Infections in the ward in which you work?

- yes
- no

If yes, who usually gives verbal information on HAIs to inpatients?

- physicians
- nurses
- other healthcare professionals (describe)_____________________________________________

Do you know whether the patients’ relatives also receive any verbal information on Healthcare-Associated Infection in the ward in which you work?

- yes
- no
- only when the patient cannot read or understand information
- only when the patients has acquired an infection

Have you ever given verbal information on healthcare workers to your inpatients?

- never
- sometimes
- usually
- always

Have you ever been asked for information on Healthcare-Associated Infections by a patient?

- never
- sometimes
- usually
- always

In your opinion, which patients should receive information on the measures to prevent Healthcare-Associated Infections?

- any inpatient
- only the inpatients at an increased risk of acquiring a healthcare-associated infection
- only the patients who are infected
- healthcare professionals should determine whether to give information to the patients on a case-by-case basis
